# Multi-objective Allocation Optimization of Soil Conservation Measures Under Data Uncertainty

**DOI:** 10.1007/s00267-023-01837-6

**Published:** 2023-05-29

**Authors:** Moritz Hildemann, Edzer Pebesma, Judith Anne Verstegen

**Affiliations:** 1 Institute for Geoinformatics, University of Münster, Heisenbergstraße 2, 48149 Münster, Germany; 2 Department of human geography and spatial planning, Utrecht University, Princetonlaan 8a, Utrecht, 3584 CS The Netherlands

**Keywords:** Spatial optimization, Conservation measure allocation, Uncertain spatial data, Stochastic objective functions, Multi-objective optimization

## Abstract

Many regions worldwide face soil loss rates that endanger future food supply. Constructing soil and water conservation measures reduces soil loss but comes with high labor costs. Multi-objective optimization allows considering both soil loss rates and labor costs, however, required spatial data contain uncertainties. Spatial data uncertainty has not been considered for allocating soil and water conservation measures. We propose a multi-objective genetic algorithm with stochastic objective functions considering uncertain soil and precipitation variables to overcome this gap. We conducted the study in three rural areas in Ethiopia. Uncertain precipitation and soil properties propagate to uncertain soil loss rates with values that range up to 14%. Uncertain soil properties complicate the classification into stable or unstable soil, which affects estimating labor requirements. The obtained labor requirement estimates range up to 15 labor days per hectare. Upon further analysis of common patterns in optimal solutions, we conclude that the results can help determine optimal final and intermediate construction stages and that the modeling and the consideration of spatial data uncertainty play a crucial role in identifying optimal solutions.

## Introduction

Securing food supply is one of the major global challenges of the present and future, and FAO (2017) considers soil erosion a main threat to meeting future food demand. Most soil erosion occurs on cultivated land being used to provide crops for subsistence use or trade. Sheet and rill erosion cause the highest soil loss on cultivated land (Hurni et al., 2016) and must be stopped or reduced to stabilize (Arora et al., 2022) or increase crop production (Gachene et al., 2020). Soil and water conservation (SWC) measures protect vulnerable areas from sheet and rill erosion (Lakew et al., 2019; Kassawmar et al., 2018; Alemu and Melesse, 2020). The question arises why SWC measures do not protect every area affected by soil loss. Several hindering factors are reported for low adoption rates of installing SWC measures (Betela and Wolka, 2021, Sileshi et al., 2019). One common reason is the high labor requirement to install and maintain the physical structures of SWC measures (Hassen et al., 2021).

Due to high labor requirements and insufficient labor, constructing conservation practices in an area is generally infeasible. By dividing an area into sub-units, in the context of soil and water conservation into sub-watersheds (Hurni et al., 2016), the required labor can be reduced by selecting a fraction of the area for treatment. The trade-off between labor or soil loss rates can be identified for every sub-watershed individually. Therefore, deciding which sub-watersheds of an area are selected for conservation ideally is a combinatorial problem that, with every additional sub-watershed, increases exponentially in complexity with 2 (selected for conservation/ not selected for conservation) to the power of the number of watersheds.

With increasing complexity, the evaluation of all possible combinations can become infeasible. (Meta-)Heuristic optimization is a method to find optimal or close to optimal solutions without evaluating all possible solutions (Yusoff et al., 2011). In comparison to single-objective optimization algorithms, multi-objective optimization algorithms find a set of optimal compromise solutions between the objectives using the principle of Pareto optimality: solutions that cannot improve in one objective without becoming worse for other objectives are Pareto optimal and referred to as non-dominated solutions (Deb et al., 2002). All Pareto optimal solutions combined form the Pareto front.

The real world can not be described by data, at least not without uncertainty (Zhang and Goodchild, 2002). Liu et al. (2007) point out that uncertainty directly influences decision-making in watershed management. One source of uncertainty that affects the planning of soil and water conservation measures is the uncertainty in spatial data required to estimate the soil loss rate or the labor cost contain uncertainties. When required input data for computing the estimates of precipitation or soil properties contain uncertainties, then the estimates become uncertain, too. Therefore, such data uncertainty should be incorporated into the optimization. Eskandari and Geiger (2009) describe a method that allows a multi-objective optimization to handle stochastic objective functions.

To the best of our knowledge, no research about optimizing the allocation of bench terraces or similar SWC measures has been conducted under uncertainty. Furthermore, no study has been conducted about multi-objective spatial optimizations applying the methods of Eskandari and Geiger (2009) to handle stochastic objective functions. The aim is to optimize the allocation of SWC measures, i.e., bench terraces, on the sub-watershed level to minimize soil and labor requirements. By considering data uncertainty, our study provides methods and information that can be used to secure future food supply in areas with high soil loss rates.

In this work, we aim to answer the following research questions: (1) How does the uncertainty of spatial input data propagate to the uncertainty in the objective values in the final Pareto fronts? (2) What common characteristics do sub-watersheds share in Pareto-optimal solutions? (3) What information can be derived from the Pareto fronts for optimal SWC measure allocation planning despite uncertainties?

## Methods

### Overview

**Fig. 1:**
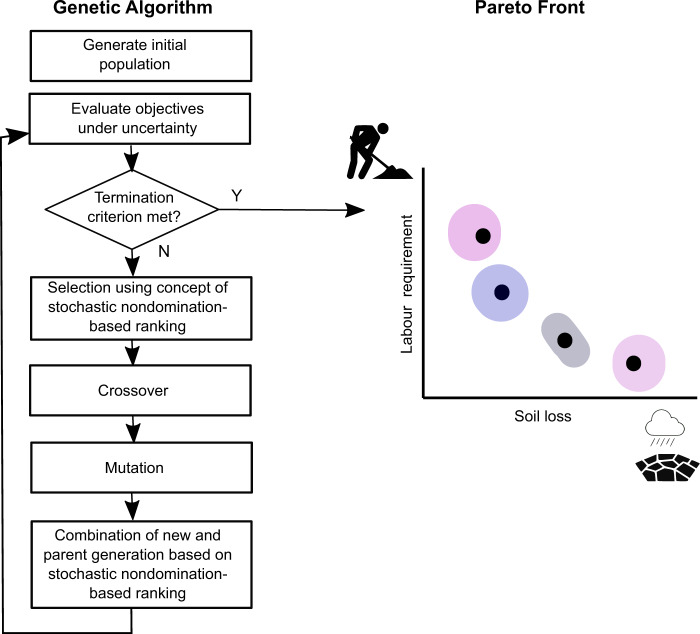
Conceptual workflow of optimizing the allocation of SWC measures

**Fig. 2:**
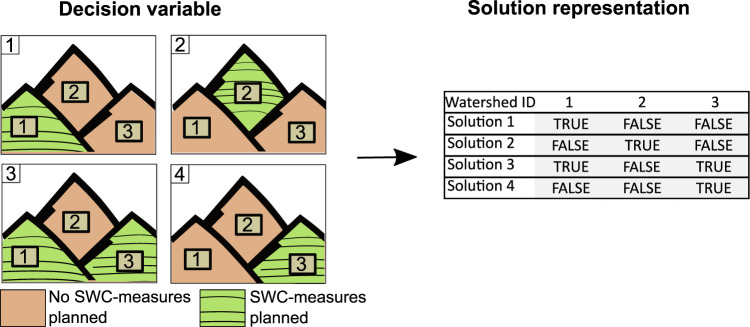
The decision variable of conserving or not conserving a land unit with soil conservation measures (left) and the solution representation in the algorithm

The designed workflow (Fig. 1) illustrates all necessary steps to optimize SWC measure allocation under uncertainty. We use sub-watersheds as decision units. In the units where the SWC measures shall be applied, bench terraces are planned (Fig. 2) with slope-dependent spacing between terraces. Each combination of units with SWC measures represents one solution of the population of the multi-objective genetic algorithm. We use the Non-dominated sorting Genetic Algorithm II, which is a common choice to optimize spatial allocation problems (Naseri et al., 2021, Shaygan et al., 2014, Strauch et al., 2019, Verstegen et al., 2017).

Realizations of spatial input data are produced with simulation methods to model spatial input data uncertainty, and each realization represents one possible outcome of the simulated variable. The realizations serve as input to the stochastic objective evaluations within the optimization after Eskandari and Geiger (2009). The stochastic objective values define the ranking of solutions for the selection and recombination procedures. After running the optimization, we analyze the Pareto fronts. We also identify solutions with soil loss rates exceeding tolerable soil losses, and we identify solutions that exceed the available labor. The results show, whether and where uncertainties in the objective values affect farmers and decision-makers.

We select three rural areas in Ethiopia from different agro-ecological zones as case studies. High soil loss rates and minimal access to labor-reducing tools or machines make them suitable examples. Hurni et al. (2016) even stated that “soil erosion is the most dangerous ecological process observed in Ethiopia, degrading the precious soil resources which are the basis of agricultural production and food for the country’s people”.

In the following sections, we explain the optimization with the representation of solutions and how solutions are evaluated (Section *SWC measure allocation optimization*). Then, the concept of multi-objective evolutionary algorithms under uncertainty is explained (Section *Multi-objective evolutionary algorithm underuncertainty*). After that, the three different case study areas are presented (Section *Case studies*). Finally, the computation of realizations from uncertain spatial input data for the stochastic objective functions is explained (Section *Simulating the spatial variables for optimization*).

### SWC Measure Allocation Optimization

#### Allocation of SWC measures as decision variable

The decision variable of the optimization is a list containing the sub-watershed identifier and the decision, and whether or not SWC measures are applied (Fig. 2). The length of the list depends on the number of sub-watersheds that varies per study area depending on the digital elevation model and the size of the area.

The study areas are separated into sub-watersheds with a watershed delineation algorithm. The selected watershed delineation is performed with a multiple flow direction model (Holmgren, 1994) using the A^T^ least-cost path search algorithm (Ehlschlager, 1989). In addition, a basin threshold parameter serves to control the minimum inflow area for sub-watersheds.

The placements of the bench terraces within each selected sub-watershed for conservation depend on the slope and the depth of workable soil. The distance between the planned bench terraces should be 2.5 times the depth of workable soil (Hurni et al., 2016). The distance between bench terraces becomes smaller with higher steepness levels and shallower soil profile depths.

#### Objective functions

***Soil loss estimation***

We use the empirical-based Revised Universal Soil Loss Equation (RUSLE) (Renard, 1997) to estimate the soil loss of protected and unprotected sub-watersheds. Even though the RUSLE only accounts for soil loss through sheet and rill erosion and not erosion types like gully erosion or dispersive soils (Rowlands, 2019), it belongs to the most widely applied methods to estimate soil loss rates (Ganasri and Ramesh, 2016). It is computed with

1 $$A=R\cdot K\cdot L\cdot S\cdot C\cdot P$$

where

A: the estimated average annual soil loss and temporal average soil loss per unit of area in t ha^−1^ yr^−1^,

R: Rainfall-runoff erosivity factor in MJ mm ha^−1^ h^−1^ yr^−1^,

K: Soil erodibility factor in (t ha h) (ha MJ mm)^−1^,

L: Slope length factor in m,

S: Slope steepness factor in radians,

C: Cover management factor (unitless),

P: Support practice factor (unitless).

The computations for the single factors of the RUSLE with study area specific parameter settings are explained in Appendix A.

***Labor requirement estimation***

We use empirical values of the labor requirements (Table 1) measured in person days from Tenge et al. (2005) for different slopes and soil types. The soil types are categorized into stable and unstable soil, where clay soil is considered stable, and loam and sand are considered unstable (Tenge et al., 2005). Soil is classified as clayey soil if the clay content is above 40%, or if the clay content is above 35% as long as the sand content is below 45% (García-Gaines and Frankenstein, 2015). The labor requirement map depends on the slope, clay, and sand content rasters (Table 1). The total labor requirement is computed for all cells with planned SWC measures:

2 $$L{D}_{total}=A\mathop{\sum }\limits_{n=1}^{N}l{d}_{n}$$

where

**Table 1:** Labor requirement estimation in labor days per hectare for building bench terraces per slope and soil classes

Slope (%)	5–12	13–25	26–35	36–55	>55
Stable soil	66	148	237	354	427
Unstable soil	92	205	328	491	592

*LD*_*total*_: total labor days

*n*: current cell of labor requirement raster

*N*: number of cells where SWC measures are applied

*A*: Cell size in ha

*ld*_*n*_: labor days per ha

***Translation of labor requirements and soil losses to monetary units***

Labor and soil losses are associated with estimated costs measurable in monetary units. We use estimated labor costs in US Dollars based on daily wages of 4.32 US Dollars after Bachewe et al. (2016). The monetary loss associated with soil loss is based on yield loss estimates, assuming an estimated yield loss of 0.74% per mm of eroded soil (Rickson, 2020). The yield loss in percent is one component of the monetary loss estimate. The second component is market shares of agricultural products, crop yields and prices derived from official statistics Central Statistical Agency Ethiopia (2020). We consider the total area used for a crop for the market share and use the cereals teff, sorghum and maize. These make up 88.52% of the total market share. For example, in the Ethiopian region Meher, 16.5% of the area is used to grow teff with a retail price of 750 US Dollars per ton in 2018 (United States Department of Agriculture Foreign Agricultural Services, 2019). In combination with crop productivity of 16.38 quintals (1 quintal = 100 kg), we obtain the expected monetary unit per ha. Both components and the total area size in hectares lead to the total estimated monetary loss in US Dollars per year. The estimate considers expected yield and soil losses for the coming 10 years.

### Multi-objective Evolutionary Algorithm under Uncertainty

#### Non-dominated sorting genetic algorithm II

We use the widely applied multi-objective evolutionary algorithm NSGA II (Deb et al., 2002) for land conservation optimization under uncertainty. The first step of the NSGA II by Deb et al. (2002) is initializing the first generation of solutions. Here, solutions to the problem are created at random. All solutions are evaluated with the two objective functions described in Sec. *Objective functions*. In the NSGA II, the solutions get assigned a non-domination rank following the following domination principle: A solution *A* is dominated by a solution *B* if all objective values of solution *A* are better than the corresponding objective values of solution *B*. The ranks indicate which solutions are non-dominated and which are dominated by other solutions. Non-dominated solutions constitute the first rank and the Pareto front. First-rank solutions dominate all other solutions, and all solutions that are only dominated by the first-rank solutions belong to the second rank. This procedure continues until all solutions have a rank. Then, a density estimation called crowding distance quantifies how similar the objective values of one solution are to the objective values of neighboring solutions in the objective space.

In the tournament selection procedure, solutions are drawn randomly from the population into a tournament pool, where the tournament pool size is a parameter. The solutions of the tournament pool are compared by their ranks. Solutions of a better rank are selected over solutions of a lower rank. If solutions are of the same rank, the solutions with higher crowing distances are selected. The selected solutions proceed to the crossover. In every crossover operation, the genes of two selected solutions (parents) are combined to produce new solutions (offspring). Random genes of produced offspring are manipulated in the mutation to encourage population diversity until the number of offspring equals the number of parents. Hereafter, the offspring population and parent generation population are merged, and the solutions with the best ranks survive. When multiple solutions have the same rank and are more numerous than the population size, the solutions with the highest crowding distances survive.

#### Stochastic nondomination-based ranking procedure

Since we propose an optimization under uncertainty, we now introduce the required adaptions to the NSGA II. Eskandari and Geiger (2009) proposed a nondomination-based ranking procedure of (Deb et al., 2002) that takes into account uncertainty in the objective values. Compared to the NSGA II, every solution has an ensemble of objective values. The selection and the recombination of the offspring and parent generation, also called survival, use the ensemble objective values. For the nondomination-based ranking procedure under uncertainty (Eskandari and Geiger, 2009), the solutions are assigned one of two ranks, the first rank with stochastically nondominated solutions and the second rank is formed by all dominated solutions. The following definition defines stochastic dominance between two solutions A and B: “Solution A stochastically dominates (is better than) solution B if ${\bar{{{{\rm{f}}}}}}_{i}(A)$ is less than ${\bar{{{{\rm{f}}}}}}_{i}(B)$ for each objective function *i*” (Eskandari and Geiger, 2009), where $\bar{{{{\rm{f}}}}}$ is the sample mean of the objective values per solution.

***First rank***

All solutions of the current generation are compared to each other. If a solution is not stochastically dominated by any other solution, it is added to the first rank. The crowding distances are computed as the fitness value for all identified solutions belonging to the first rank.

***Second rank***

The second rank combines all solutions dominated by the first-rank solutions. For the second-rank solutions, the summation of the probabilities that a solution dominates other solutions is computed, referred to as expected strength values *E*_*S*_. To compute *E*_*S*_, we define amongst all second-rank solutions whether or not a solution A dominates or is dominated under uncertainty by another solution B, where the following statement defines dominance under uncertainty: “Solution A significantly dominates (is better than) solution B with a confidence level of […](1 − *α*) if ${\bar{{{{\rm{f}}}}}}_{i}(A)+hwi$(A) $< {\bar{{{{\rm{f}}}}}}_{i}(B)-hwi$(B) for each objective function i” (Eskandari and Geiger, 2009), where ${\bar{{{{\rm{f}}}}}}_{i}$(x) − *hwi*(x) and ${\bar{{{{\rm{f}}}}}}_{i}$(x) + *hwi*(x) are the lower and upper bounds of the objective value interval at significance level *α*. Then, the probabilistic dominance *P* is computed with three possible cases of *P* (this definition holds only for minimization problems):

1. The probabilistic dominance *P* of a solution A over solution B is 0 when all lower bounds of A are higher than the upper bounds of B.
2. The probabilistic dominance *P* of a solution A over solution B is 1 when all upper bounds of A are lower than the lower bounds of B.
3. If case 1 and 2 both do not apply, the probabilistic dominance *P* of a solution A over solution B is a certain probability *P*_*A*_ when all lower bounds of A are less than the corresponding upper bounds of B.

The probability *P*_*A*_ that objective values of solution A are lower than the objective values of B is computed with the following equation, which approximates the integral *Q*(*x*) using the suggestion of Borjesson and Sundberg (1979):

3 $$P(A \,<\, B)=1-Q\left(\frac{{\mu }_{B}-{\mu }_{A}}{\sqrt{{\sigma }_{A}^{2}+{\sigma }_{B}^{2}}}\right)$$

4 $$Q(x)=\frac{1}{2}{{{\rm{erf}}}}\left(\frac{x}{\sqrt{2}}\right).$$

where

*μ* : mean of objective values,

*σ* : standard deviation of objective values,

erf(*x*) : Gaussian error function

The summed-up probabilities *P* values of every solution in the second rank of dominating the other solutions in the second rank (number of solutions are the same per generation) result in the expected strength value *E*_*S*_. Lastly, we calculate the fitness value *E*_*F*_ for each solution in the second rank. For a solution A, the *E*_*F*_ is the sum of all *E*_*S*_ values solutions by which solution A is stochastically dominated minus the sum of all *E*_*S*_ values solution A stochastically dominates.

#### Selection and survival under uncertainty

For the selection and survival under uncertainty, we use the ranks and computed fitness values *E*_*F*_ for the tournament selection (Sec. *Non-dominated sorting genetic algorithm II*). We use the tournament selection with a tournament pool size of two (binary tournament selection). Compared to the selection without uncertainty, the *E*_*F*_ are considered when two second-rank solutions are compared: If only one solution is of the first rank, it wins. If both solutions are of the first rank, the solution with the higher crowding distance wins. If both solutions are of the second rank, the solution with the higher *E*_*F*_ wins. The crossover and mutations produce offspring with the operators of the NSGA II. After that, the survival of solutions from the combined population of parents and offspring takes place. The ranks and fitness values are recomputed for the combined population. The crowding distance defines the order of the first-rank solutions, and the *E*_*F*_ values define the second-rank order. The best solutions are retrieved from the ordered population until meeting the population size limit.

#### Seeding

We extend the described multi-objective optimization under uncertainty by seeding. Seeding is the injection of elite solutions into the initial population. We follow the method of Guariso and Sangiorgio (2020), who found that seeding the single objective optimal solutions benefits the spread and convergence of the Pareto fronts. Hildemann and Verstegen (2021) found that the findings hold for a multi-objective land use allocation optimization under uncertainty using the NSGA II. Therefore, the single objective extreme solutions are computed and injected into the initial population: The single objective extreme solution for minimizing the soil loss rates is to have every sub-watershed selected. The single objective extreme solution for minimizing the labor requirement is to omit SWC installations completely.

### Case Studies

The case study areas are three Kebeles, the smallest administrative districts in Ethiopia, named Gumobila, Enerata, and Mender 51. In all three study areas, the depth to bedrock exceeds 104 cm (Hengl et al., 2015), which allows the equidistance between bench terraces to be set as 5 m (Hurni et al., 2016). The basin threshold parameter for the watershed delineation, i.e., the minimum area of a watershed, is set to an equivalent of 0.2 acres. Using this parameter, the watershed delineation results in 147 sub-watersheds in Gumobila, 137 in Enerata, and 47 in Mender 51.

**Fig. 3:**
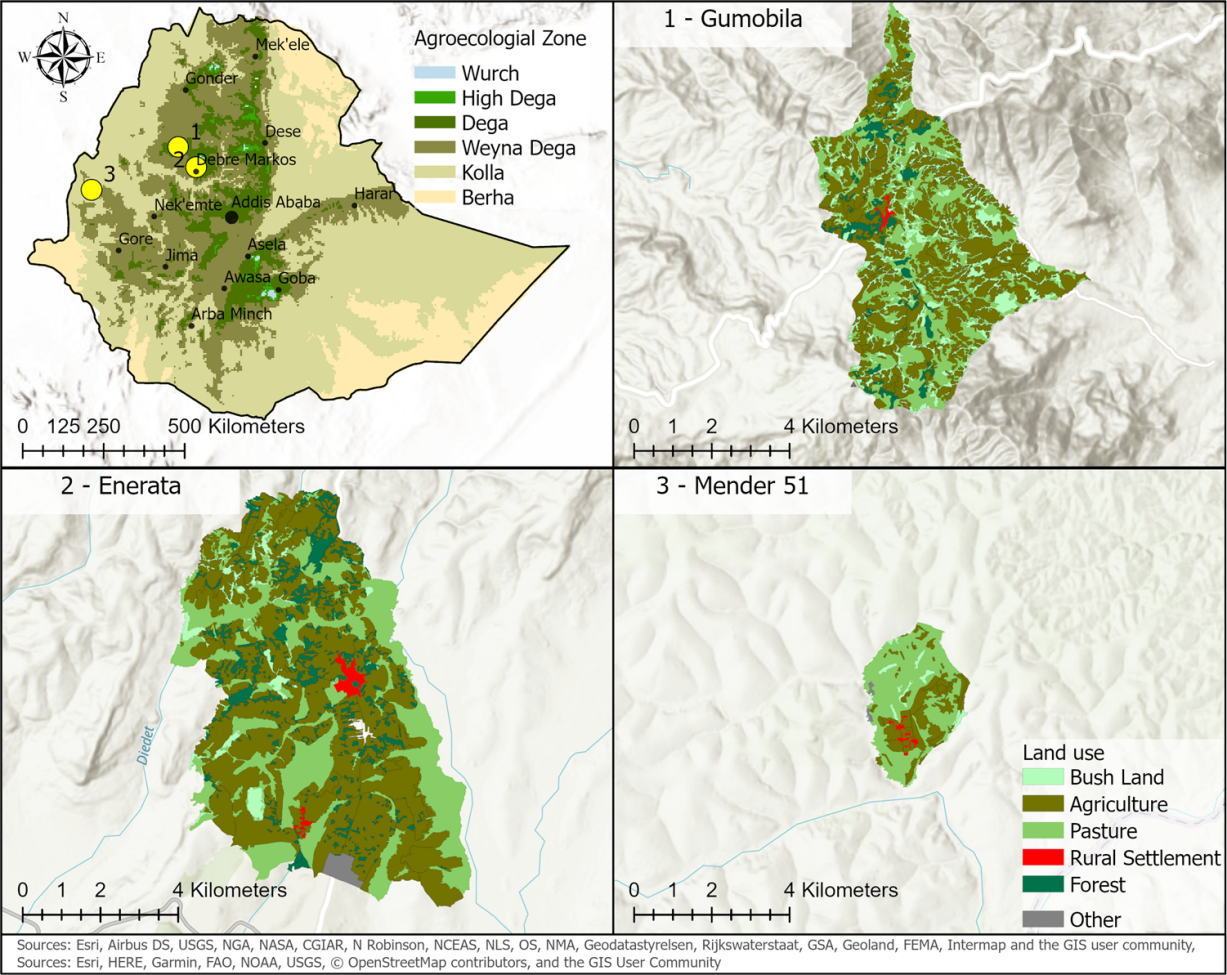
Locations and land use of selected study areas

The case study areas were selected because each Kebele is in a different agro-ecological zone with high soil loss rates (Hurni et al., 2016), and because the Kebeles are all rural areas, with most farmers being subsistence farmers. Furthermore, land use information was made available for these Kebeles by Deutsche Gesellschaft für internationale Zusammenarbeit (GIZ) GmbH (2021). More than 80% of the land of the selected areas is used for cereal production with sorghum, teff and maize as main crops (Central Statistical Agency Ethiopia, 2020). The Kebeles are located in the north-western part of Ethiopia (Fig. 3). The Kebeles Gumobila and Enerata belong to the West Gojjam zone in the Amhara region, Kebele Mender 51 belongs to the Asosa zone in the Benishangul-Gumuz region.

The altitude of Gumobila (Fig. 3) ranges from 2048 to 3106 m above sea level with a mean annual rainfall of 1970 mm. Gumobila belongs to the agro-ecological zone Wet Dega. The second study area Enerata (Fig. 3) has an altitude between 2283 m and 2638 m above zero with mean annual rainfall of 1305 mm, situated in the agro-ecological zone called Moist Dega. The most western study area Kebele Mender 51 (Fig. 3), is classified as Wet Kolla with an altitude between 1335 m and 1478 m above zero and a mean annual rainfall of 1780 mm.

We computed the estimated total available labor per hectare with the number of households, the area size and national statistics (Central Statistical Agency Ethiopia, 2016) to set the required labor from optimal solutions in relation to the available labor from the local population (Table 2). The estimated available labor for SWC measures varies from 54 to 69.2 labor days per hectare (LD/ha).

**Table 2:** Estimated maximum available labor per hectare based on number of households from Deutsche Gesellschaft für internationale Zusammenarbeit (GIZ) GmbH (2021) and statistics about the average number of persons per household from Central Statistical Agency Ethiopia (2016) and the assumption of 220 working days per year and a maximum dedication of work time of 40% to the installation of SWC measures

Study area	Nr. of households	Estimated agricultural area in ha per person	Estimated available labor for SWC measures in LD/ha
Gumobila	890	1.62	54
Enerata	4961	1.33	66
Mender 51	1556	1.27	69.2

### Simulating the Spatial Variables for Optimization

In optimizations ignoring data uncertainty, all variables are presumed to be accurate. In comparison, optimizations under uncertainty incorporate variables with their associated uncertainty. In this work, we simulate possible outcomes following distribution functions of uncertain variables. The following explains how we simulate uncertainty for modeled and observed spatial data. The realizations serve as inputs to both objective functions. The uncertain precipitation data affects the rainfall-runoff erosivity factor (R) of the RUSLE (Sec. *SWC measure allocation optimization*). The uncertain soil properties affect the soil erodibility factor (K) of the RUSLE. Both factors and associated uncertainties affect the soil loss estimation. Furthermore, the uncertain soil properties affect the classification into stable clayey and unstable loamy and sandy soil, which affects the labor requirement estimation.

#### Simulating soil variables under uncertainty

The required soil variables are bulk density, sand, silt and clay fractions, and organic matter fractions. Those variables are provided in the global soil dataset called SoilGrids (Poggio et al., 2021). We select this dataset because few soil data samples are available in the global soil sample database WoSIS (Batjes et al., 2020) for Ethiopia with the required variables. The modelled soil variables by Poggio et al. (2021) use soil samples in combination with auxiliary variables to predict the soil variables with machine learning models at a resolution of 250 meters. The median and lower and upper limits of a 90% prediction interval from a tenfold cross-validation are available for multiple soil depths.

We apply the moving average model (Haining, 1978) to generate the soil variables. The method allows generating realizations with a spatial auto-correlation resembling the auto-correlation of the SoilGrid variables. We want to point out that this auto-correlation could be an artifact from the machine learning predictions rather than the auto-correlation in the soil variables. To apply the moving average model, we use the reported median and prediction intervals of soil variables provided by SoilGrids. Before the moving window average is applied in Eq. (7), random values *X* are drawn based on:

5 $$X \sim {{{\mathcal{N}}}}(\mu ,{\sigma }^{2})$$

6 $$\sigma =\sqrt{N}\frac{({P}_{95}-{P}_{5})}{2t}$$

where

*μ* : Median of modeled soil variables,

*N* : Sample size, in this case 10 due to tenfold cross-validation,

*t* : t value of for *p* = 0.05 and *N* − 1 degrees of freedom (1.833)

*X* : Independently drawn value from the normal distribution,

*P*_5,95_ : Lower and upper limits of a 90% prediction interval.

The moving average model smoothes *X* and does not act as an exact interpolator (Cressie, 2015). The neighborhood needs to be defined for the smoothing, either by a fixed euclidian distance or by the k nearest neighbors. We define the neighborhood as all cells within the neighborhood range *M* of 4 pixels.

7 $$S(X)=\frac{1}{{(2M+1)}^{2}}\mathop{\sum }\limits_{j,k=-M}^{M}{X}_{[j,k]}$$

where

*M* : Neighborhood range (here: 4).

Afterwards, a min-max scaling operation is performed for the silt, clay and sand fractions to ensure that all fractions lie between 0% and 100% and sum up to 100%.

#### Simulating precipitation under uncertainty

We use the CHIRPS global rainfall data from 1981 to 2021 from Funk et al. (2015) as precipitation data. The data has a spatial resolution of ~5.5 km. Three to six grid cells of the precipitation data cover each study area (Fig. 3). Without adaptation, the large grid cell size results in assumed precipitation input without spatial variability or discrete borders of the natural continuous precipitation variable between grid cells.

In order to simulate precipitation on a finer resolution, we apply a top-kriging approach (Skøien et al., 2006). The top-kriging approach allows predicting a variable with quantified uncertainty in which observations are areas instead of points. This approach enables interpolation from large areas to smaller areas. We use CHIRPS data with an area 100 times larger than the target study areas and apply top-kriging for a target resolution of 100 m at the study areas. The output from the top-kriging approach is the predicted precipitation at the finer target resolution.

After that, we perform a conditional simulation to create realizations with the same spatial dependence as the sample precipitation data. We use conditioning by kriging simulation (Chiles and Delfiner, 1999). The idea behind the conditional simulation is to condition a random field with the kriging estimator. The first step of the conditioning by kriging simulation is to perform a non-conditional simulation to produce a random field. Here we use the turning bands method (Chiles and Delfiner, 1999). In a second step, the random field from the non-conditional simulation *S*(*x*), which must have the same co-variance as the sample data, is then conditioned by the Kriging estimator Z^*^(*x*) following equation (8).

8 $$T(x)={Z}^{* }(x)+[S(x)-{S}^{* }(x)]$$

where

*x* : Data points,

*T*(*x*) : Conditional simulation,

Z^*^(*x*) : Kriging estimator,

*S*(*x*) : Non-conditional simulation,

S^*^(*x*) : Kriging estimator of *S*(*x*) with the variogram function and observed data of Z^*^(*x*).

Due to the conditioning by kriging, every realization *T*(*x*) has the same degree of spatial dependence as the estimated spatial dependence of the sample precipitation data and obeys the spatial pattern in the sample data. The same spatial dependency can not be achieved with just the non-conditional simulations.

#### Design of simulation experiment

We execute three simulation experiments that build on one another.

The first experiment is about choosing the required number of realizations for the stochastic objective functions. For this purpose, we analyze the objective value distributions of a reference solution with an increasing number of realizations. When the objective values stabilize, we assume that the number is sufficient to evaluate solutions despite uncertainty. The reference solution is a solution in which SWC measures are applied in every second sub-watershed. We select the reference solution since it is a trade-off solution between both objectives.

We evaluate the proposed optimization algorithm performance in the second simulation experiment to see whether it can converge to the true optimum. Since computing all possible combinations is infeasible for the whole study area, we define a subset area with a small number of sub-watersheds. Ten sub-watersheds of the study area Gumobila with 1024 possible solutions for this benchmarking serve this purpose. This low number of possible combinations allows enumeration (Galluccio et al., 2001) to identify all optimal solutions deterministically, forming the true Pareto front. This true Pareto front is the benchmark to evaluate the proposed algorithm’s performance. Due to the highly decreased problem complexity, a small population size of 40 and 30 generations suffices for the algorithm evaluation. The comparison allows estimating how many solution evaluations are required until the optimization converges to the true Pareto front.

In the third simulation experiment, we execute the optimizations for the three study areas from different agro-ecological zones with a population size of 100 and 200 generations. The resulting Pareto fronts contain the optimal solutions for minimizing soil loss rates and labor requirements.

### Implementation

The conditional simulation of the precipitation was performed in R (R Core Team, 2017) with the packages rtop (Skøien et al., 2014) for the kriging, RandomFields (Schlather et al., 2015) for the unconditional simulations, and the spatial data packages sp (Bivand et al., 2013), sf (Pebesma, 2018), raster (Hijmans, 2021) and rgdal (Bivand et al., 2021) for GIS operations. For the soil data simulations, Python 3 (van Rossum and Drake, 2009) was used with the packages numpy (Harris et al., 2020) and scipy (Virtanen et al., 2020). As optimization algorithm, the Python package pymoo (Blank and Deb, 2020) was used and adapted. The loss estimations (RUSLE) were performed with QGIS (QGIS Development Team, 2009) and GRASS (GRASS Development Team, 2017) with multiprocessing. The Python packages matplotlib (Hunter, 2007) and interactive visualization package plotly (Plotly Technologies Inc., 2015) were used for the visualizations. All used software is open source software, and the implementation is fully reproducible (Dataset with DOI will be linked here). The study is designed to be executable on a Windows computer with 16 GB RAM, i7-9850H Intel Processor with 6 cores and 12 logical processors.

## Results and Discussion

### Simulated Data

**Fig. 4:**
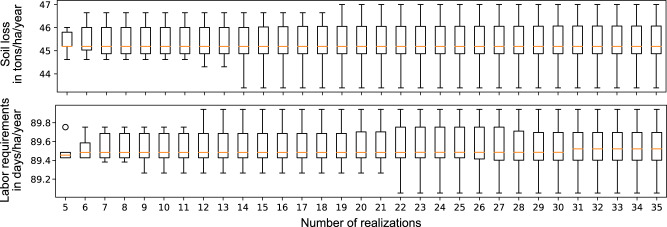
Simulated spatial input data under uncertainty and the histograms for 5–35 realizations for the study area Gumobila

The two objective value distributions from the estimated soil loss rates and labor requirements obtained with the reference solution remain stable after 19 and 22 realizations for the study area Gumobila (Fig. 4). The distributions also remained stable with less than 22 realizations in the other two study areas. Therefore, we chose the highest number of 22 realizations for all study areas.

**Fig. 5:**
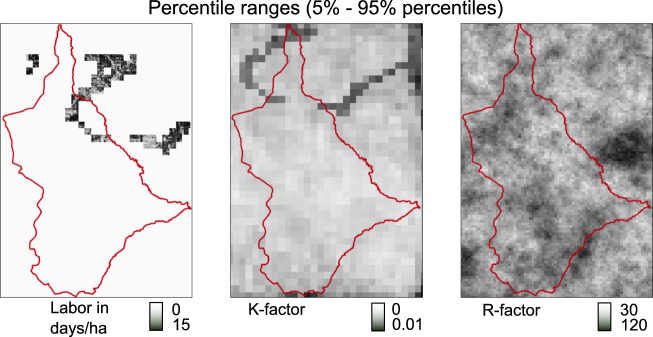
Spatial distribution of the simulated spatial input data under uncertainty illustrated with the percentile range (5%,95%) for the soil erodibility factors K, the labor requirement and rainfall-runoff erosivity factors R

**Fig. 6:**
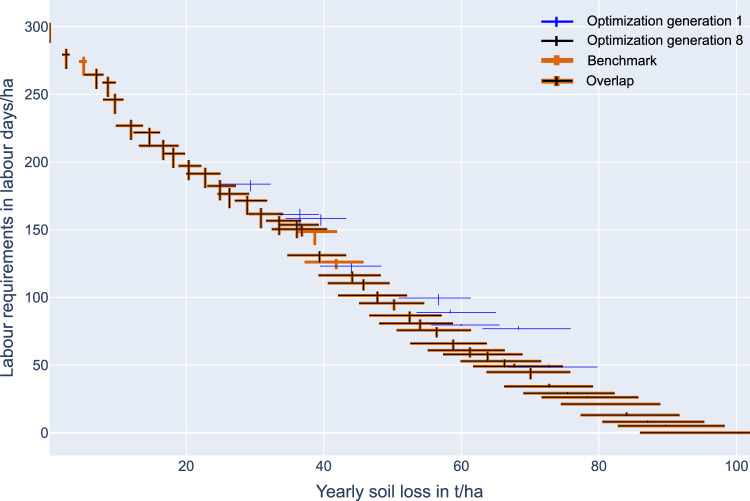
Convergence of Pareto front of optimization with population size 40 to Pareto front from the deterministic evaluation on a subset of 10 sub-watersheds for study area Gumobila. The crosses illustrate the maximum range of the uncertain objective values per solution, the uncertainty in the labor requirement is illustrated by the vertical lines, the uncertainty in the soil loss rates is illustrated by the horizontal lines. The expected monetary loss estimation is described in Sec. *SWC measure allocation optimization*)

The differences between the 5% and 95% percentiles are relatively evenly distributed over space for the R-factor realizations. In contrast, the differences in labor requirements from the K-factor realizations (factors of RUSLE, Sec. *SWC measure allocation optimization*) are more localized in the northern parts of the study area (Fig. 5). Only 2.5% of the study area shows differences in the estimated labor requirements. This is caused by the low occurrence of loamy or sandy soil fractions and predominantly high clayey soil fractions in the study areas. Since clayey soil is considered stable, only a small part of the 2.5% of the area is further classified as unstable (Table 1). Due to the even more clayey soil in the study area Mender 51, no uncertainty was observed in the labor requirements Fig. 6.

### Benchmark

For the benchmark, we evaluated all possible 1024 solutions. The benchmark Pareto front from all possible solutions resulted in 43 Pareto-optimal solutions. The optimization resulted in 40 solutions, and the comparison with the benchmark Pareto shows that all 40 solutions are true Pareto optimal solutions. Moreover, only 8 generations with just 320 solution evaluations were required. Therefore, the proposed optimization can find all the true optimal solutions with 68.75% fewer solution evaluations for the Gumobila study area. However, this does not guarantee that the optimization can find the true optimal solutions for the whole study area with more sub-watersheds. Still, the simulation experiment proves the proposed optimization can find the true optimal Pareto front.

### Pareto Fronts

**Fig. 7:**
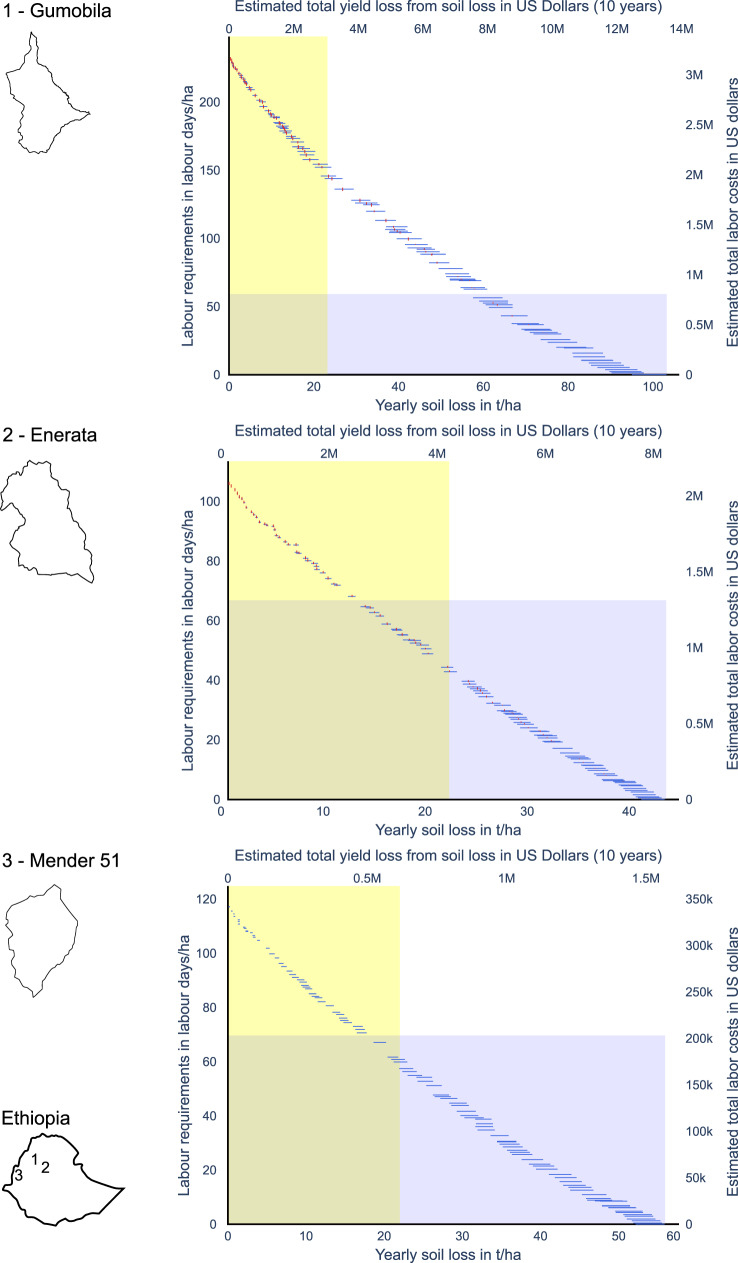
Pareto fronts with the labor requirements in LD/ha (*y*-axis, left) and in US Dollars (*y*-axis, right), and the estimated yearly soil loss in t/ha/yr (*x*-axis, bottom) and estimated yield loss in US Dollars (*x*-axis, top). The light yellow region illustrates the tolerable soil loss rates after Hurni (1983), and the light blue region illustrates the estimated available labor days by farmers of the study areas (Table 2). Vertical red lines and horizontal blue lines illustrate maximum objective value ranges due to uncertainty

The different Pareto fronts of the three study areas (Fig. 7) reveal substantial differences: The maximum labor requirements are 105 LD/ha in Enerata, 119 LD/ha in Mender 51 and 230 LD/ha in Gumobila. Correspondingly, the maximum yearly soil loss rates are almost 240% higher in Gumobila, with 100 t/ha/yr, compared to Enerata with 42 t/ha/yr. As a consequence, it is possible to obtain tolerable soil loss rates for Ethiopian soils of 22 t/ha/yr (Hurni, 1983) in Mender 51 with less effort compared to Enerata and Gumobila and the costs per hectare (in LD) are 210% and 350% lower, respectively. The differences in rainfall regime and slopes mainly cause the differences: On average, Mender 51 and Enerata have a slope of 5.3° and 7.2°. In contrast, Gumobila has the highest average slopes of the study areas being 16.4°, resulting in higher erosion estimates based on the RUSLE. Furthermore, Gumobila also has the highest yearly precipitation of the three study areas. Consequently, the estimated total yield losses per study area vary a lot. When estimating the monetary loss associated with the obtained yield losses, the results show that in Mender 51 the monetary loss is 1.5 million USD, whereas the estimated monetary loss can exceed 14 million USD in Gumobila. The high difference in yield loss in monetary terms is not only caused by different maximum soil loss rates but also by the different study area sizes. The relations to reduce soil loss rates per added labor are most similar. Over the whole Pareto front, on average, 1 ton of yearly soil loss per hectare can be prevented by providing the required labor of 2.5 LD/ha in Gumobila, 2.55 LD/ha in Enerata and 2.1 LD/ha in Mender 51.

In Gumobila, the estimated soil loss rate is 6 t/ha/yr (5.8%), in Enerata it is 2.3 t/ha/yr (5.3%), and in Mender 51, it is 3 t/ha/yr (5.2%). The uncertainties for the estimated required labor objective values are relatively small, with 2.5 LD/ha (1%) in Gumobila, 0.8 LD/ha (0.07%) in Enerata, and 0 in Mender 51. Furthermore, a clear trend of uncertainty can be observed in the objective values over the Pareto fronts: the relationships between the mean objective values and the uncertainty of the objective values remain stable. The uncertainties make up, on average, 11.6% (Gumobila), 4.9% (Enerata), and 5.4% (Mender) of the mean of the soil loss rates. Correspondingly, the ranges make up 0.3%, 0.2%, and 0% of the mean labor requirements. Therefore, solutions with low mean objective values of one objective show low uncertainty for that objective and high uncertainty for the second objective, and vice versa.

For the two study areas Enerata and Mender 51, the tolerable soil loss rates for Ethiopia with 22 tonnes per hectare can be achieved with the estimated available labor of the local population (Table 2). For the study area Gumobila, the soil loss rates are only reducible to a yearly soil loss of only 62 tonnes per hectare with the available labor from the local population. Therefore, the highest dedication of labor in Gumobila still leads to severe soil loss after Tsegaye and Bharti (2021). In Gumobila, the estimated total amount of money to reach the tolerable soil loss rates of 22 t/ha/yr with additional (potentially external) labor is 1.1 million US Dollars.

### Locations of Conservation Measures

**Fig. 8:**
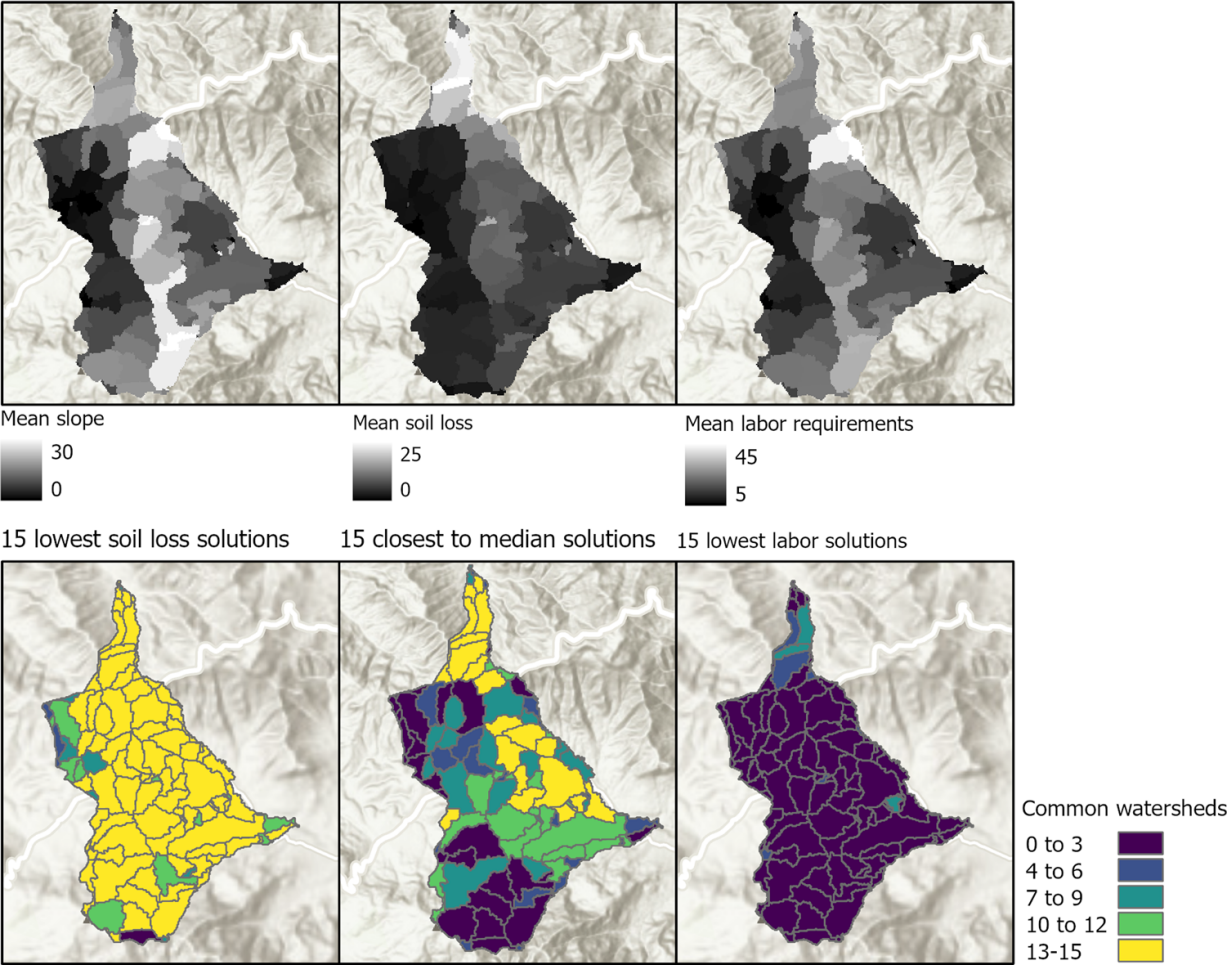
Top: Mean slope per sub-watershed, mean soil loss rate per sub-watershed without SWC measures, mean labor requirements per sub-watershed for installing SWC measures. Bottom: Number of common sub-watersheds being selected for conservation for 15 solutions with the lowest soil loss and highest labor requirement, 15 solutions closest to the median solution, and 15 solutions with the highest soil loss and lowest labor requirement

In addition to deriving the Pareto fronts, we identify common characteristics of the non-dominated solutions. We identify sub-watersheds that are part of multiple solutions that are next to each other in the Pareto front. For this purpose, we define a neighborhood of one solution as the seven nearest solutions on either side in the Pareto front, resulting in a total neighborhood size of 15. In this context, we use the terms ‘commonly selected sub-watersheds for conservation’ for sub-watersheds that are selected by the majority of optimal solutions for conservation. Correspondingly, ‘occasionally selected sub-watersheds’ refer to sub-watersheds that are not being selected for conservation by the majority of optimal solutions for conservation within neighboring solutions of the Pareto front. Three different parts of the Pareto front of the study area Gumobila are selected (Fig. 8, bottom row), one including the 15 solutions with the lowest soil loss rates, one including the 15 solutions with the lowest required labor, and one neighborhood that agglomerates the 15 solutions related to the median soil loss rate solution of the Pareto front. For example, the two sub-watersheds with the highest mean soil loss rates in Gumobila are commonly protected by SWC measures even in the 15 solutions with the least labor. On the other hand, sub-watersheds with high labor requirements, e.g., in North East, are only occasionally selected for conservation. This holds for the 15 solutions with the least required labor and also for the 15 solutions surrounding the median soil loss rate solution. Furthermore, two patterns stand out in the 15 solutions surrounding the median soil loss solution (Fig. 8, bottom row, middle): Firstly, almost every sub-watershed in the northern part with mean soil loss rates above 21 t/ha/yr is selected for conservation across optimal solutions, even though the labor requirements are high with 27–31 LD/ha. This observation indicates that the conservation of sub-watersheds with the highest soil loss rates is important to for a solution to be identified optimal regardless of high labor costs. Secondly, the sub-watersheds in the middle-eastern part of the study area with moderate soil loss rates of 5–11 t/ha/yr and moderate labor requirements of 15–23 LD/ha are commonly selected for conservation. All sub-watersheds with low mean soil loss rates are only occasionally selected for conservation; only 11% of the sub-watersheds with soil loss rates below 5 t/ha/yr are selected for conservation more than 7 times out of 15. This indicates that sub-watersheds with low mean soil loss are selected for conservation in the optimal solutions if the labour requirements are close to the minimum.

**Fig. 9:**
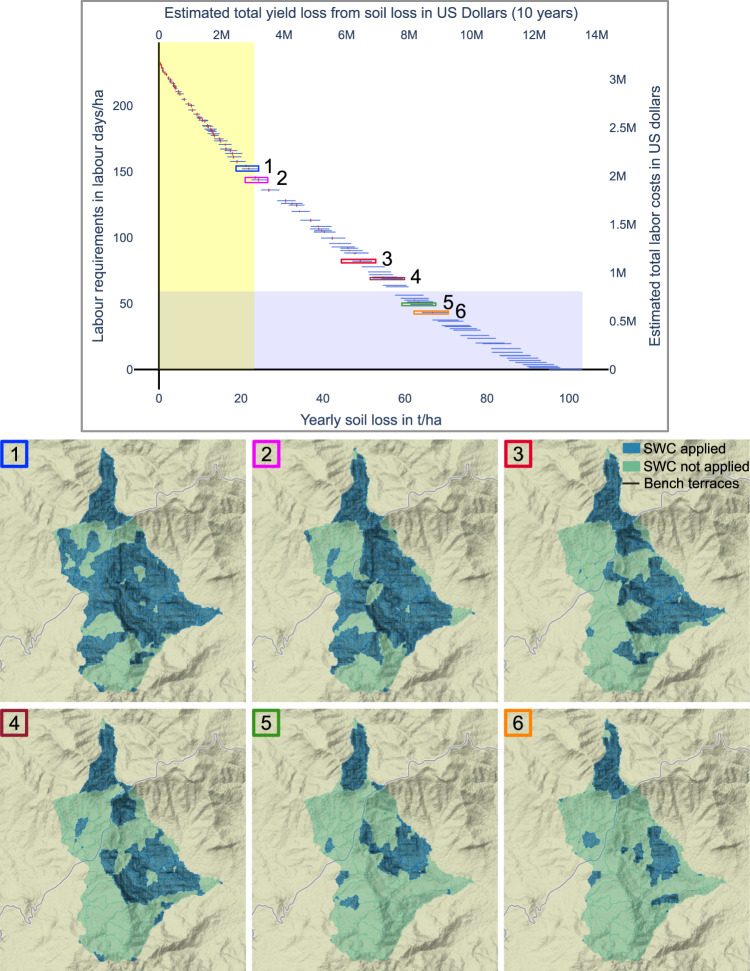
Selected solutions with their position in the Pareto front and the selected sub-watersheds for conservation with bench terraces

Furthermore, we observe robust patterns in the selected sub-watersheds for conservation in optimal solutions, illustrated by six representative solutions (Fig. 9). Here, we use the term solution robustness (Ales and Elloumi, 2021), which refers to structural similarities of solutions instead of similarities between objective values. In this context, robust solutions are optimal solutions that can be modified easily according to a change in the environment (Tjornfelt-Jensen and Hansen, 1999) where modified solutions are still of high quality. Therefore, a solution is robust if other solutions with mostly the same sub-watersheds are selected for conservation. When analyzing the proportion of common sub-watersheds in the example solutions along the Pareto front (Fig. 9), we can see that, on average, 76% of the selected sub-watersheds for conservation in solution 6 (Fig. 9) are also selected for conservation in the solutions 1–5. There are just a few sub-watersheds selected for conservation in solution 6 with the highest soil losses that are not selected for conservation in solutions 1–5. This behavior is observable in most solutions for all three study areas. Identifying the commonly selected sub-watersheds for conservation along the Pareto front can lead to a temporal order for ongoing SWC measure implementations. If the following scenario was considered: a specific solution within the tolerable soil loss region of the Pareto front is aimed for in the long term, in this example solution 6, with insufficient labor to develop all the structures. In such a case, the robust sub-watersheds being also selected for conservation in other optimal solutions with lower labor requirements can be identified and developed first. The missing sub-watersheds ordered in priority by the mean soil loss rates can be developed in consecutive years. This approach ensures that the final implementation state is optimal and that the intermediate implementation states are optimal, or at least close to optimal. The same benefits apply in a second scenario in which conservation goals become more ambitious over time. Selecting an optimal solution with lower soil loss rates with the same robust sub-watersheds to be selected for conservation allows further developing one optimal solution into another optimal solution.

We provide an interactive visualization tool^1^ for an in-depth inspection of the solution space. The tool intends to simplify the investigation of the Pareto front and display selected solutions without requiring a full understanding of the underlying optimization procedure.

### Uncertain Consequences for Local Population from Spatial Data Uncertainty

The estimated consequences for the local population, in terms of what soil loss rates they face and how much labor they would need to invest, are more uncertain in specific parts of the study area. This propagates to the Pareto fronts, both in the soil loss and labor requirement objective values.

Fewer sub-watersheds being selected for conservation through SWC measures result in higher estimated soil losses and uncertainties of the soil loss (Fig. 7). The reason for the higher soil loss uncertainty is the much higher uncertainty in sub-watersheds without conservation: protected watersheds have low or no estimated soil loss. Therefore, solutions with higher proportions of sub-watersheds not-selected for conservation have higher uncertainty. Over the whole study area, the uncertainty within the soil loss objective values varies is 5.8%. This means the estimated yield loss over 10 years can differ by ~700,000 USD for the whole study area simply due to uncertain soil loss rates. In sub-watersheds in the north of Gumobila, the soil loss rates vary up to 14%, caused by unevenly distributed input data uncertainties. This range is similar to identified yield loss risks by droughts from Leng and Hall (2019). Therefore, the worst-case scenario from the considered spatial data uncertainty can considerably threaten subsistence farmers.

The uncertain labor requirements are strongly clustered in space. The reason for this spatial cluster is the uncertainty in the classification into stable and unstable soil: Stable clayey soil leads to lower labor requirement estimates than unstable loamy or sandy soil. Due to the predominantly high clayey soil fractions in the study areas, only a small fraction of the total area is classifiable into stable and unstable soil with the given uncertain spatial data. For this reason, all uncertainty within the labor requirement objective values originates from only 2.5% of the total study areas. While most sub-watersheds of the study area are not affected by the spatial data uncertainty, the range is up to 15 LD/ha in the small fraction of the total study area (Fig. 5), and only half of the optimal solutions have uncertain labor requirement objective values.

Furthermore, Fig. 9 illustrates how much additional labor is required to reduce the soil loss rates of a specific solution. For example, to avoid the estimated soil erosion rates of solution 6, solution 4 can be implemented. Implementing the SWC measures of solution 6 requires additional labor input of 15 LD/ha compared to implementing the SWC measures for solution 4. With the agricultural area per farmer of 1.62 ha, the average single farmer would need to invest 24 additional labor days.

### Identified Challenges to Implement Optimal Solutions

So far, we have discussed theoretical solutions to the given problem. The required actions from the local population for installing SWC measures are diverse and labor intensive. Therefore, the challenges for the actual implementation of optimal solutions need to be discussed, too.

The total labor made available by the local population (Table 2, light blue areas in Fig. 7) can not be presupposed to be met unquestioned since the presumed dedication of 40% of the work time from all the male population between 15 and 59 years old to install SWC measures (Table1) is ambitious to achieve. The practical hindrances for plowing due to terrace construction, the transport of construction material and tools, and the sacrifice of even minor parts of the scarce production areas in Ethiopia to areas covered by bench terrace constructions hinder planned implementations. The hindrances increase with steeper slopes with shorter distances between bench terraces (Schiechti, 1985). Another complication is to convince farmers to assist in protecting land with conservation measures they do not own. Ethiopian farmers do not own land under the current land tenure policy, and the land property rights are expected to remain public (Crewett et al., 2008). Teshome et al. (2016) and Kagoya et al. (2018) state that insecure land tenure is one of the main factors decreasing the SWC measure adoptions by farmers. Also, the benefits and costs of installing the SWC measures must be distributed among the farmers of the study area. If the soil of a sub-watershed with installed SWC measures is selected for conservation with labor from other farmers, there needs to be an exchange for the labor. Since subsistence production accounts for 58% of the agriculture in Ethiopia (Sibhatu and Qaim, 2017), the ability of farmers to pay workers is unlikely.

Governmental subsidies are an option to encourage farmers. Mekuriaw et al. (2018) showed that regions with governmental support for land conservation have twice as high SWC measure adoption rates compared to regions without that support. Driving factors are knowledge provision about the consequences and technical and financial support. Providing tools and/or additional labor and financing may be required to implement optimal solutions. Such support may even be cheaper than the tax losses from the long-term consequences of the estimated total yield losses. Moreover, conservation measures help to protect the soil as the fundamental basis for agricultural production. Conservation becomes especially important regarding the reported severe hunger threats (IPC, 2021) in parts of the Ethiopian rural population and increasing population rates without viable non-agricultural income opportunities.

### Limitations and Future Work

In this work, we considered data uncertainty related to precipitation and soil data. The considered uncertainty may be modeled differently and be extended in future work. We used discrete classes of stable or unstable soil depending on the clay and sand content. Combined with the slope, these classes result in a discrete labor requirement assessment. The results showed that these classes lead to highly localized differences in labor days within just 2.5% of the study area (Fig. 5). The real relationship between labor requirements and workable soil is most likely more complex and potentially continuous. A more detailed labor requirement estimate under various soil and slope conditions could reduce the highly localized labor requirement uncertainty.

Furthermore, the uncertainty of the temporal variables can be taken into consideration. Spatio-temporal modeling requires all input data to be available for multiple timestamps, which is currently not available: Even though future climate projections can help to consider the temporal uncertainty of the precipitation, the temporal development of the labor requirement can currently not be modeled. Modeling the temporal uncertainty of the labor costs requires two currently unavailable datasets: The spatial distribution of access to tools and machines in Ethiopia and a socio-economic temporal trend of the access. Currently, the only information available is that <1% of the Ethiopian population has access to mechanized tools in agriculture (Ayele, 2022). For this reason, we excluded the temporal uncertainty in this work.

We only consider the detachment of material but not the transport and deposition, which may have additional effects, such as contaminant transport (He et al., 2009). Sediment yields from soil erosion (Endalew and Biru, 2022) can be even beneficial under specific circumstances (Stern et al., 2020). If certain areas are too steep to be cultivated or used for other purposes, the lost soil is less critical. If the soil or the sediments of the soil is transported by the downstream flows to agricultural land and deposited sediments may be used to re-stock damaged soils in productive areas. If, on the other hand, contaminants associated with agricultural production (Endalew and Biru, 2022) are transported, scarce fresh or even drinking water reservoirs can be contaminated (Singh et al., 2022). Due to the low level of fertilization in rural areas of Ethiopia, such contamination might not be severe yet, but it might be in the future. In addition, the effects of sediment transport can be modeled under uncertainty: The used clay, silt, and loam fractions can be used to estimate sediment transport, even under different climate change scenarios (Maruffi et al., 2022). A modified objective function that considers the positive and negative effects of sediment transport in allocating conservation measures yields interesting future work, with and without uncertainty modeling.

Furthermore, we use the total soil loss as the sole metric and don’t consider the depth or quality of the lost soil. In future applications, it would be interesting to include further metrics that consider the depth of the topsoil layer or organic carbon contents, which is a key aspect of soil quality (van Beek et al., 2019). These metrics can also define the temporal order of implementing the conservation measures: depending on the relative gain in productivity, protecting fragile topsoils before areas with more robust topsoils, or the reverse, may help to maintain maximum agricultural productivity. Such temporal prioritization can help to prevent scenarios in which soil degradation is so severe that any potential to regenerate soils (Schreefel et al., 2020) is lost, and it can be coupled with the temporal prioritization under uncertainty. The incorporation of such metrics in the allocation of conservation measures comes with difficulties for prioritizing: Is it more important to prioritize fragile and thin topsoil layers to protect near-future agriculture in those regions, or is it more important to prioritize topsoil layers that potentially yield more stable crop yields in the long-term?

Another limitation is that the spatial uncertainty of the digital elevation model (DEM) was not considered. This uncertainty could lead to uncertain borders of the sub-watersheds, as the results of Aerts et al. (2003) indicate. In their case, the DEM uncertainty propagated to different ski courses, indicating that DEM uncertainty could propagate to different borders of the sub-watersheds. In the proposed optimization algorithm, the uncertain distinction between sub-watersheds leads to solutions with uncertain decision variable definitions: The number of decision variables can change (Hildemann and Verstegen, 2021), and the reference of a sub-watershed identifier to the area becomes ambiguous. Since the imposed difficulties and problem complexity would increase greatly, we did not consider that uncertainty in this study. However, it is interesting future work in a more theoretical context for spatial optimizations with uncertain extents of the decision variables.

Another possible future research is to extend the decision variable of the optimization. One option is to consider more SWC measures, e.g., to combine allocating bench terraces with the planning of dams (Xu et al., 2012), rehabilitation areas or waterways as suggested by Hurni et al. (2016). Another approach would be an extended approach in which SWC measure allocation is coupled with land use allocation optimization. In that case, the land use would not serve as input as in the current optimization set-up. Instead, the land use allocation is optimized in a first step for a set of objectives. Then, optimal land use can be used as input for the SWC measure allocation. This approach yields the benefit of further reducing soil loss rates.

## Conclusion

In this work, we optimized the allocation of SWC measures for the objectives of soil loss rate minimization and labor requirements minimization under spatial data uncertainty in three Ethiopian rural areas. We modeled uncertain soil and precipitation variables and used them for stochastic objective value evaluations.

Our first research question was how the uncertainty of spatial input data propagates to the uncertainty in the objective values in the final Pareto fronts. In the study area Gumobila, the highest range in the estimated soil loss rate objective function from uncertain spatial data is 6.0 t/ha/yr (5.8% of corresponding mean soil loss), in study area Enerata it is 2.3 t/ha/yr (5.3%) and in study area Mender 51 it is 3 t/ha/yr (5.2%).

Our second research question addressed what shared characteristics of sub-watersheds can be observed in optimal solutions across the Pareto front. Optimal solutions share the characteristic that sub-watersheds with the highest average soil loss rates are most often selected for conservation, regardless of high labor costs. Furthermore, optimal solutions share the characteristic that sub-watersheds with low mean soil loss are rarely selected for conservation unless the labour requirements are close to the minimum.

Our third research question was what information could be derived from the Pareto fronts for optimal SWC measure allocation planning despite data uncertainties. We observed the following pattern in six representative optimal solutions: Most of the selected watersheds for conservation in the solutions with the highest soil loss rates were also selected for conservation in the other five solutions with lower soil loss rates. This observation allows the identification of optimal final implementation states while the intermediate implementation states are also optimal or close to optimal.

We conclude that SWC measure allocation optimization supports the identification of optimal final and intermediate SWC construction states and that the consideration and modeling of spatial data uncertainty plays a crucial role in the identification of Pareto optimal solutions.

## Appendix

### Revised universal soil loss equation

The rainfall erosivity factor R was computed using Hurni’s equation which is adapted to an empirical analysis in Ethiopia (Hurni, 1985):

A1 $$R=-8.12+(0.562\cdot p)$$

where

*p* : Precipitation in mm yr^−1^.

The soil erodibility factor K expresses the susceptibility of a soil to erode which can be calculated with soil properties such as organic matter content, soil texture, soil structure and permeability (Panagos et al., 2014).

A2 $$K=\frac{2.1\cdot 1{0}^{4}\cdot {M}^{1.14}(12-OM)+3.25\cdot (s-2)+2.5\cdot (p-3)}{100}\cdot 0.1317$$

where

*M* : textural factor computed as (silt fraction + very fine sand fraction) ⋅ (100 - clay fraction),

*O**M* : Organic matter fraction,

*s* : soil structure class (1: very fine granular, 2: fine granular, 3: medium or coarse granular, 4: blocky, platy or massive) derived with silt, clay and sand fraction s and the corresponding USDA soil textural classes (García-Gaines and Frankenstein, 2015).

The water path flow length and the water speed define the kinetic energy of the water on the soil surface, and are expressed by the slope length (L-factor) and slope steepness (S-factor). Wischmeier and Smith (1978) defined the L-factor as the ratio of soil lost from a horizontal slope length to the corresponding loss from the slope length.

A3 $$L={\frac{\lambda }{22.13}}^{m}$$

A4 $$m=\frac{\beta }{\beta +1}$$

A5 $$\beta =\frac{\frac{\sin (\theta )}{0.0896}}{0.56+3\sin {(\theta )}^{0.8}}$$

where

*θ* : slope angle in degrees,

*m* : ratio of rill and interrill erosion calculated with *β*.

The S-factor is computed with empirical equations for different slope levels to express the relation between soil loss and slope steepness (Renard, 1997).

A6 $$S=\left\{\begin{array}{ll}10.8\cdot s+0.03,\quad &s \,<\, 0.15708\\ 16.8\cdot s+0.5,\quad &s > =0.15708\end{array}\right.$$

where

*θ* : slope angle in degrees,

*s* : slope angle in radians.

The cover management factor C and supporting practices factor P express the relationship of land cover and erosion measure application to the soil erodibility. The C- and P-factors (Table 3) differ per land and base on empirics for Ethiopia from Zerihun et al. (2018) and Kebede et al. (2021). Kebede et al. (2021) define different P-factor values for cropland with and without the physical SWC measure Fanya Juu. Fanya Juu is a conservation measure applied in Ethiopia which shall progress into a bench terrace in the long term.

**Table 3:** Cover management (C) and supporting practices (P) factors per land use for Ethiopia

Land use	C-factors	P-factors
Agriculture with conservation measures, slope ≤ 15%	0.5	0.325
Agriculture with conservation measures, slope > 15%	0.5	0.356
Agriculture without conservation measures	0.5	1
Pasture with conservation measures	0.2	0.7
Pasture without conservation measures	0.2	1
Forest	0.001	1
Shrubs	1	0.2
Water	1	1
Urban	0.011	1

## Footnotes

1 https://github.com/mohildemann/visualization-landconservation-optimization
